# Effects of the Ratio of Substituting Mineral Fertilizers with Manure Nitrogen on Soil Properties and Vegetable Yields in China: A Meta-Analysis

**DOI:** 10.3390/plants12040964

**Published:** 2023-02-20

**Authors:** Shaobo Wang, Rui Lv, Xinhua Yin, Puyu Feng, Kelin Hu

**Affiliations:** 1 Key Laboratory of Arable Land Conservation (North China), Ministry of Agriculture and Rural Affairs, College of Land Science and Technology, China Agricultural University, Beijing 100193, China; 2 Department of Plant Sciences, University of Tennessee, Knoxville, TN 37996, USA

**Keywords:** manure nitrogen substitution, soil organic carbon, soil total nitrogen, soil-available nutrients, vegetable yield

## Abstract

Substituting mineral fertilizers (MFs) with manure nitrogen (N) can not only reduce environmental pollution, but also improve soil quality. However, the effects of various manure N substitution ratios (SRs, the ratio of manure N over total N applied) on soil properties and vegetable yields in China are poorly studied. Here, through a meta-analysis of 667 observations, we assessed the effects of three manure N SRs (low (SR ≤ 35%), medium (35% < SR ≤ 70%), and high (SR > 70%)) on vegetable yields and soil properties (soil organic carbon, SOC; soil total nitrogen, STN; microbial biomass carbon (C) and nitrogen (N), MBC/N; and available phosphorus and potassium, (AP/AK)) in the 0–20 cm soil under different climatic conditions, initial soil properties, and management practices. The results show that the SOC and STN contents increased by 28.5% and 21.9%, respectively, under the medium SRs compared to the MF, which were the highest among the three SRs. Both soil MBC and MBN increased with the increase in the SRs, and the increased ratios in the high SRs reached 203.4% and 119.3%, respectively. In addition, the AP also increased with the increase in the SR, but the AK was not significantly changed with the low and medium SRs compared with the MF. Overall, the medium SR produced the highest vegetable yield among the three SRs with an increase of 18.6%. Additionally, a random forest analysis indicated that the N application rate, planting years, and mean annual precipitation were the most important factors influencing vegetable yield. In conclusion, the SR of 35–70% is more conducive to increasing soil nutrient contents significantly and improves vegetable yields in Chinese vegetable fields.

## 1. Introduction

In China, more than 4.0 billion tons of livestock and poultry manure are produced each year, and about 78% of the N from livestock excreta is lost to the environment, which results in a waste of resources and serious environmental pollution [1,2,3]. Converting livestock and poultry manure into manure nitrogen (N) and then using it to substitute mineral fertilizers (MFs) can improve soil fertility, remediate degraded soils, enhance crop yields, and stabilize cropland ecosystems [4]. China is a major producer of vegetables, and its planting area of vegetable accounts for 13% of the total cropping area [5]. The input of MF in vegetable fields in China increased 2.1 times from 6 million tons in 1998 to 12.9 million tons in 2014. The large-scale application of MF not only leads to a decline of vegetable quality, but also causes a series of ecological and environmental problems, such as greenhouse gas emission, soil acidification, and water pollution [6]. In order to alleviate this contradiction, the substitution of MFs with manure N has gradually been accepted by vegetable growers in recent years and has become an important aspect of sustainable vegetable production. However, some researchers have found that when the substitution ratio (SR) of manure N is too low, the soil cannot provide sufficient available nutrients for vegetable production, which significantly affects vegetable yields and fails to improve soil quality [7]. If the SRs are too high, this usually leads to an excessive accumulation of nutrients in the soil, increases leaching of dissolved organic nitrogen (DON) and carbon (DOC), and results in adverse effects on the soil, such as the accumulation of antibiotics and heavy metals [8,9]. Therefore, an appropriate SR of manure N is of great significance to maintain the stability of vegetable production and the sustainable utilization of livestock and poultry manure resources.

There is a consensus among researchers around the world on the synergistic effects of adding manure N to mineral sources to maintain both micro- and macronutrient supplies for crops [10] and improve the efficiency of mineral fertilizers while producing longer-term benefits for soil fertility [11,12]. China has adopted many measures during the past 20 years to enhance nitrogen use efficiency (NUE) and reduce environmental risks, including the soil testing and fertilizer recommendation (STFR) project (MOA, 2005); soil organic matter enhancement project (MOA, 2012); and the Organic-Substitute-Chemical-Fertilizer (OSCF) action (MOA, 2017) [2]. Manure N has been recognized as the most suitable substitute of MF, and manure N application can increase nutrient contents (including soil organic carbon (SOC), soil total N (STN) [13,14], microbial biomass C and N(MBC/MBN) [15,16], and available phosphorus (AP) and potassium (AK) [17]). Manure also contains S, C, and micronutrients, and the accumulated nutrient elements in the soil can affect soil fertility and plant growth [18,19]. Manure N application has been considered as an appealing practice to enhance the SOC content from the perspectives of both agronomic production and mitigating global climate warming [20,21]. Giacometti et al. [22] found that the SOC and STN contents were increased by 34% and 31%, respectively, in cow dung treatments compared to the MF in a 46-year long-term experiment. Xin et al. [23] reported that compared with the initial soil, the SOC level increased by 4.0 and 2.3 times after 23 years of the manure N SR being 100% and 50%, respectively, relative to the MF. Qaswar et al. [24] conducted a manure N experiment for 34 years and showed that, compared with the application of MFs, when the SR was 50%, the STN, AP, and AK increased by 22%, 150%, and 70%, respectively. However, when the SR was 100%, the AP and AK decreased by 10% and 3%, respectively. In southern Italy, the application of mineral fertilizers led to soil acidification and reduced soil microbiota diversity, activity, and functionality, with negative effects on crop yield, while manure N effectively improved soil fertility and promoted the development of a beneficial soil microbiota [25]. Liang et al. [26] evaluated the results of a 17-year fertilization experiment and showed that soil MBC and MBN increased by 31.7% and 50%, respectively, with an SR of 50% compared with the MF. The above results indicated the positive effects of manure N on soil fertility, and these results are important for each individual study, but different SRs cause large differences in nutrient availability. Thus, it is essential to determine the reasonable SRs of manure N in large areas, which can provide useful guidance for the application of manure N.

The effect of manure N on crop yield is still controversial. The responses of yield to substituting manure for fertilizer may vary with the proportion of manure N substitution [27]. Xia et al. [28] conducted a global meta-analysis of 141 studies and found that substituting manure N for mineral fertilizers (with an equivalent N rate) significantly increased the crop yield by 4.4% and significantly decreased environmental pollution. Pincus et al. [29] conducted farm studies on highly weathered soils in the Lake Victoria Crescent of Uganda and found that the highest yields were obtained when the fertilizer was applied at a ratio of 67% manure N to 33% mineral fertilizer. Cai et al. [4] reported that the application of manure N in acidic soils increased the content of SOC and soil nutrients and had a positive effect on crop yield. A meta-analysis of 153 experiments in China revealed that manure N application increased crop yield by about 8.5–14.2 Mg ha^−1^ [30]. However, it has also been reported that the yield could not be improved due to the slow mineralization of N in manure N and the difficulty in being absorbed by crops in a short time [31]. Especially in the process of vegetable production, the growth period of vegetables is shorter than that of other field crops, and it is difficult to synchronize crop N demand with soil N supply [6]. The level of soil nutrients directly affects the sustainability of soil productivity, which will indirectly affect crop yield levels [4,32]. Different SRs have different nutrient availability to the soil and thus exert different effects on crop yield. At present, few studies have analyzed the correlation between soil nutrient indicators and yield under the conditions of different SRs. In addition, because different SRs will also cause differences in yields due to different climatic conditions, initial soil properties, and management practices [12,33], it is warranted to comprehensively evaluate the impacts of different SRs involved in the above factors on crop yields. This helps us understand the impact of the efficient use of organic N resources in agricultural management strategies on global vegetable production.

However, traditional single-experiment studies cannot well reflect the overall response of SR effects on soil properties and crop yields, which have certain limitations in guiding farmers to adjust fertilization patterns and policy formulation. Unlike other research methods, a meta-analysis is a quantitative method that systematically integrates the results of multiple experiments, and it has been widely used in agricultural research [17,33,34], which has the advantage of being able to identify trends that may not have been detected by a single experiment. Thus, the aims of this study were (i) to evaluate the changes of soil properties in vegetable fields with different SRs by meta-analysis; (ii) to analyze the effects of climatic conditions, initial soil properties, and management practices on vegetable yields under different SRs; and (iii) to clarify the relationship between each nutrient index and crop yield and determine the appropriate SR in vegetable fields in China.

## 2. Results

### 2.1. Effect of Manure N Substitution Ratio on Vegetable Yield

**Figure 1:**
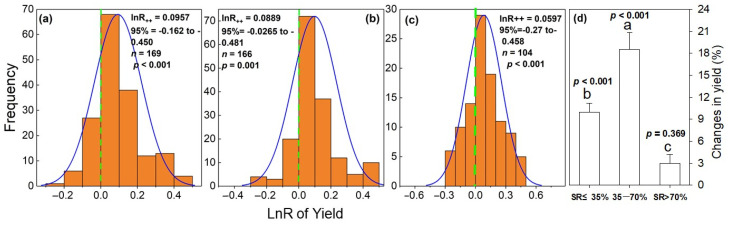
Frequency distributions of response ratios (lnR) for vegetable yield response to manure N substitution ratio (SR) compared with mineral fertilizers (MFs) (**a**–**c**) and the percentage of increase in vegetable yield (**d**). *p*-values indicate statistical significance of the overall effect size of each practice. a, Low SR, SR ≤ 35%; b, Medium SR, 35% < SR ≤ 70% and c, High SR, SR > 70%.

The lnR_++_ was normally distributed for each SR (Figure 1a–c). The normal distribution ranges of the lnR of the vegetable yield effect value were −0.162~0.450 (*p* < 0.001), −0.0265~0.481 (*p* = 0.001), and −0.27~0.458 (*p* < 0.001) for the low SR, medium SR, and high SR, respectively, and the yield variation ranges were −14.9~56.8%, −2.6~61.7%, and −23.6~58.0%, respectively. In particular, compared with the MF, the manure N substitution increased vegetable yields, and the average effect sizes under the low, medium, and high SRs were 0.095, 0.170, and 0.029, respectively (Figure 1), and the vegetable yields increased by 9.9%, 18.6%, and 3.0%, respectively (Figure 1d).

**Figure 2:**
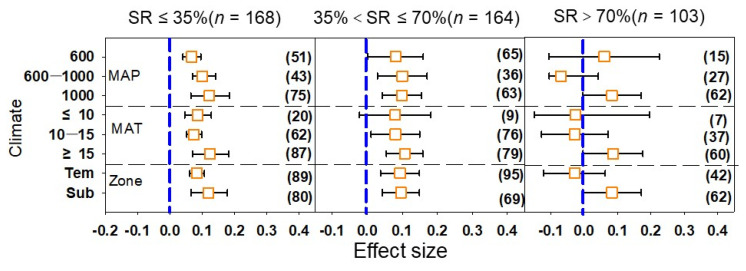
Effect of manure N substitution ratio (SR) on vegetable yields with different climatic factors. Note: MAP, mean annual precipitation (mm); MAT, mean annual temperature (°C). Tem, temperate climatic zone; and Sub, subtropical climatic zone. Blue dashed line indicates effect size is 0. The numbers in parentheses correspond to the sample size of the statistical analysis.

Among the climatic factors (Figure 2), the heterogeneity (Qb) among the SRs was small, and the difference was not significant (*p* > 0.05) (Table 1); this indicates that the different climatic factors had no significant effect on the vegetable yield. When the MAP < 600 mm, the average effect value of the vegetable yield increased with the increase in SR; however, when the MAP ranged from 600 mm to 1000 mm, the effect sizes of the medium SR and high SR were the largest and smallest, respectively. The effect value of the medium SR was the largest, followed by that of the low SR under the three MAT conditions, and the high SR had a negative value under MAT < 15 °C, which indicated that the high SR showed a trend of decreasing vegetable yield. Vegetable yields in subtropical regions were higher than those in temperate regions under each SR, and the effect value was the largest in the medium SR.

**Figure 3:**
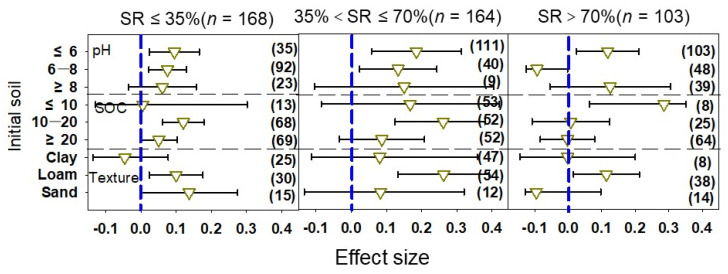
Effect of manure N substitution ratio (SR) on vegetable yield with different initial soil properties. The numbers in parentheses correspond to the sample size of the statistical analysis.

For the initial soil properties, there was moderate heterogeneity (Qb) among the SRs, and there was a significant difference among the SRs (*p* < 0.05) (Table 1); this indicates that soil properties significantly affect vegetable yield. Under acidic soil conditions (pH ≤ 6), the vegetable yield increased (Figure 3). Under neutral soil conditions (pH = 6–8), the yield increased the most in the medium SR, while the yield decreased with the high SR. When the initial soil pH was ≥8, the effect values of the low, medium, and high SRs were 0.061, 0.150, and 0.167, respectively, and the vegetable yield increased gradually with the increase in the SR. When the initial SOC was <10 g kg^−1^, the vegetable yield gradually increased with the increase in SR, but when the SOC was ≥10 g kg^−1^, the effect value of the medium SR was the largest, while the effect value was negative in the high SR. In clay soils, the vegetable yields with low and high SRs showed a decreasing trend, while under silty soils, all three SRs significantly increased vegetable yields, among which the medium SR increased yields the most. In sandy soils, the effect values of the low, medium, and high SRs were 0.137, 0.083, and −0.095, respectively, and the effect value of the yield was the largest under the low SR.

**Figure 4:**
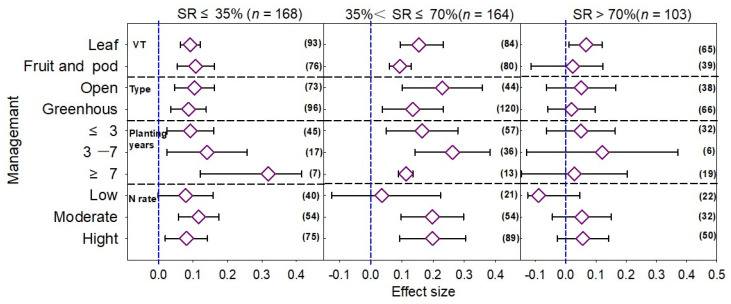
Effect of manure N substitution ratio (SR) on vegetable yield with different management practices. Note: VT, vegetable type; Type, cultivation type. The numbers in parentheses correspond to the sample size of the statistical analysis.

The yield of leafy vegetables was significantly increased by all three SRs (Figure 4), and the effect size under the medium SR was the largest at 0.155. The effect sizes on fruits and pod vegetables under the low and medium SRs were 0.107 and 0.093, respectively, but the difference was not significant in the high SR. In the open-air vegetable cultivation conditions, manure N substitution could increase vegetable yield, and the effect value of the open-air cultivation conditions was significantly higher than that of the greenhouse cultivation (Figure 4). The effect values of the low, medium, and high SRs were 0.104, 0.230, and 0.051, respectively, which showed that the medium SR had the largest effect value. However, in greenhouse vegetables, compared with a single application of MF, the effect values of the low and medium SRs were 0.087 and 0.0135, respectively, and the difference was not significant under the high SR. The effect size of the medium SR was the largest whether it was open-air or greenhouse cultivation. When the planting was <3 years, the effect value of the high SR was negative, and in 3–7 years, the vegetable yield gradually increased with the increase in SR. However, in >7 years, the increase in vegetable yield gradually decreased with the increase in SR. Under the low N application rate, the average effect values of the low, medium, and high SRs were 0.079, 0.036, and −0.089, respectively. The increase in vegetable yields showed that low > medium > high SR. The SR increase could significantly enhance vegetable yields under the medium N application rate, and the increase rate in the medium SR had the largest effect on the yield. The difference in the increase rate of high SR on vegetable yield under the high N application rate was not significant.

### 2.2. Total Effect of Manure N Substitution Ratio on Soil Properties in Vegetable Fields

**Figure 5:**
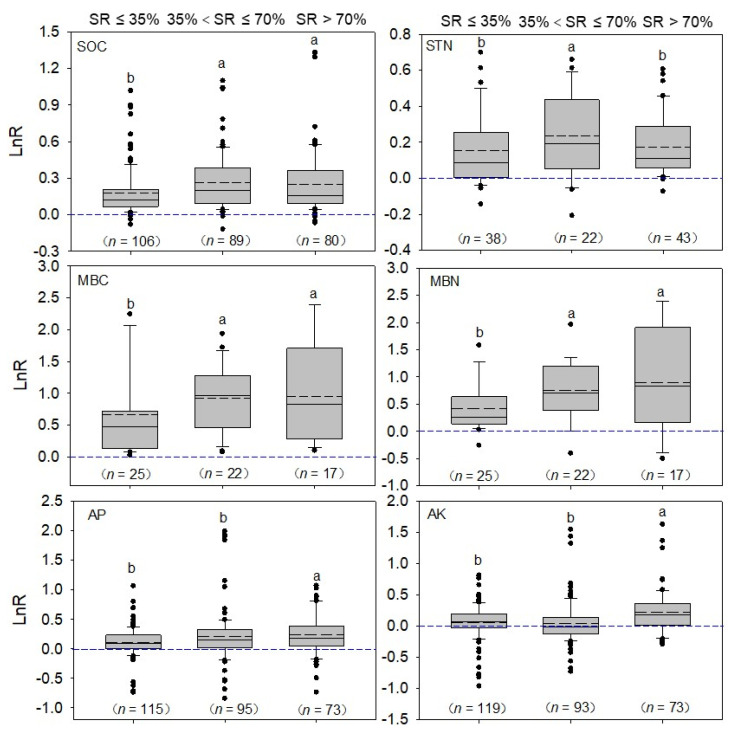
Box plot for attendant effect sizes changes in SOC, STN, MBC, MBN, AP, and AK under different manure N SRs compared with mineral fertilizer. The dotted line and solid black line indicate median and means, respectively, and circles indicate outliers. Different letters indicate that the three manure N SRs are significantly different. The number of data points is given below each box. The blue dashed line indicates that the effect value is zero.

The soil properties of vegetable fields in China are shown in Figure 5. The mean effect sizes of the medium SR of SOC and STN were significantly higher than those with the low SR, and the LnR of SOC and STN ranked as medium > high > low. Compared with those with low SR, the comparative tallness of the box plots for the high and medium SRs indicated that the LnR values of the MBC and MBN showed relatively wider distributions (Figure 5). The mean effect sizes of the medium and high SRs of MBC and MBN were significantly higher than those with the low SR (0.554). The effect sizes for MBC increased by 100.4% and 72.3% for the medium (0.955) and high (1.110) SRs, respectively, when compared to those with the low SR (0.564). The effect sizes for MBN increased by 14.0% and 39.2% for the medium (0.643) and high (0.785) SRs, respectively, when compared to the low SR. The mean effect sizes of the high SRs of AP and AK were significantly higher than those of the low and medium SRs. Therefore, only analyzing the difference between the average values cannot reflect the effects of different SRs on soil properties, which needs to be further analyzed.

### 2.3. Effect of Manure N Substitution Ratio on Soil C and N in Vegetable Fields

**Figure 6:**
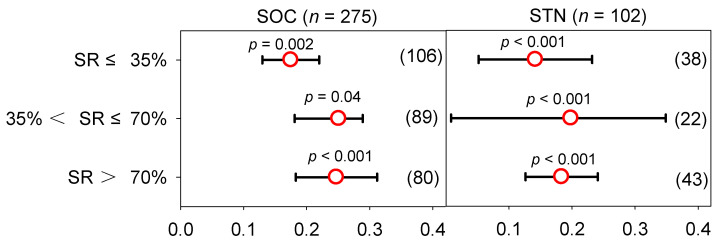
Response ratio of SOC and STN under different manure N substitution ratios (SRs). The numbers in parentheses correspond to the sample size of the statistical analysis.

For changes in SOC and STN, there was little heterogeneity among SRs (Table S1). The Qb was less than 35, and this suggests that the heterogeneity among treatments is acceptable. Significant differences were observed among different subgroups (*p* < 0.05), and this indicates that the manure N fertilizers significantly affect SOC and STN. Manure N substitution was able to increase the SOC content regardless of the climatic factors, initial soil properties, and management practices (Figure S1). The mean SOC contents in the MF and low, medium, and high SRs were 10.7, 15.1, 16.3, and 16.4 g kg^−1^, respectively (Table S2). In general, compared with the MF, the effect sizes of the low, medium, and high SRs were 0.175 (95% *CI*: 0.130–0.220), 0.251 (95% *CI*: 0.181–0.289), and 0.247 (95% *CI*: 0.183–0.311), respectively. The rank of the average effect size was as follows: medium > high > low SR. The SOC content in the low, medium, and high SRs increased with the increase in planting years (Figure S1). The mean STN contents in the MF and the low, medium, and high SRs were 1.1, 1.3, 1.8, and 1.4 g kg^−1^, respectively (Table S2). The STN showed greater heterogeneity in the low SR, and the difference among the SRs was not significant (Table S1). Except for that with the low SR, the Qb of the medium and high SR were less than 35. At the same time, the low and medium SRs were affected by climatic factors, initial soil properties, and management practices (Figure S2). Overall, compared with the MF, the effect sizes of the low, medium, and high SRs were 0.142 (95% *CI*: 0.052–0.232), 0.198 (95% *CI*: 0.008–0.349), and 0.184 (95% *CI*: 0.126–0.241), respectively. The average effect size was ranked as follows: medium > high > low SR. The above results show that the SOC and STN contents increased the most in the medium SR, and the increased ratios reached 28.5% and 21.9%, respectively (Figure 6). During the planting years of less than 7 years, the STN content increased with the increase in the SR. At greater than 7 years, the STN content gradually decreased as the SR increased (Figure S2).

**Figure 7:**
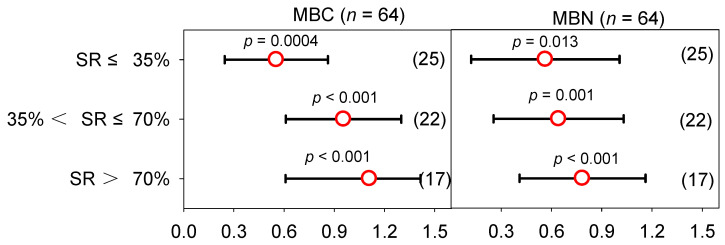
Response ratio of MBC and MBN under different manure N substitution ratios (SR).

There was little heterogeneity among the SRs (Table S1), and significant differences were detected among different subgroups (*p* < 0.05) in MBC and MBN (Figures S3 and S4). The Qb was less than 35, and this suggests that the heterogeneity among the treatments is acceptable. The mean MBC contents in the MF and the low, medium, and high SRs were 19.1, 29.6, 78.4, and 100.3 mg kg^−1^, respectively (Table S2). Overall, in the MBC content (Figure 7), compared with the MF, the effect sizes of the low, medium, and high SRs were 0.554 (95% *CI*: 0.246–0.862), 0.955 (95% *CI*: 0.610–1.130), and 1.110 (95% *CI*: 0.609–1.415), respectively. The mean MBN contents in the MF and the low, medium, and high SRs were 8.5, 15.9, 25.3, and 29.7 mg kg^−1^, respectively (Table S2). The effect sizes of the low, medium, and high SRs of the MBN content were 0.564 (95% *CI*: 0.121–1.001), 0.643 (95% *CI*: 0.254–1.031), and 0.785 (95% *CI*: 0.409–1.162), respectively. Both the MBC and MBN increased with the increase in the SR, and the increase in MBC and MBN in the high SR reached 203.4% and 119.3%, respectively. In addition, MBC and MBN also increased as the number of planting years increased (Figures S3 and S4).

### 2.4. Effect of Manure N Substitution Ratio on Available Phosphorus and Potassium in Vegetable Fields

**Figure 8:**
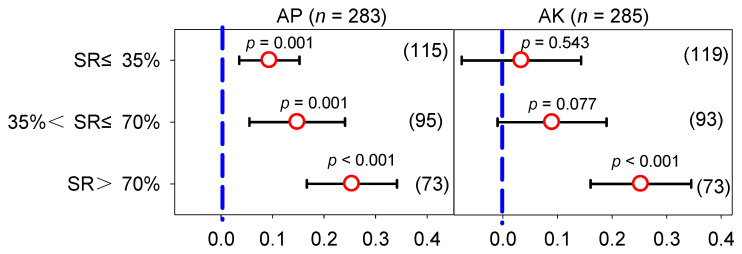
Response ratio of AP and AK under different manure N substitution ratios (SRs). The numbers in parentheses correspond to the sample size of the statistical analysis.

There was less heterogeneity (Qb < 35) in the soil AP and AK under the SRs (Table S1); Qb was less than 35, and the heterogeneity was affected by climatic factors, initial soil properties, and management practices at the low and medium SRs (Figures S5 and S6). The mean AP contents in the MF and the low, medium, and high SRs were 115.0, 138.6, 139.4, and 137.8 mg kg^−1^, respectively (Table S2). In general, compared with the application of the MF (Figure 8), the effect sizes of the low, medium, and high SRs were 0.094 (95% *CI*: 0.036–0.152), 0.148 (95% *CI*: 0.056–0.241), and 0.254 (95% *CI*: 0.167–0.342), respectively. With the increase in the SR, the soil AP gradually increased, and the average effect value of the three SRs showed as follows: high > medium > low SR. The AP also increased as the number of planting years increased (Figure S5). For the soil AK, the mean AK contents in the MF and the low, medium, and high SRs were 241.0, 245.6, 266.9, and 260.3 mg kg^−1^, respectively (Table S2); the effect sizes were 0.034(95% *CI*: −0.077–0.143), 0.090 (95% *CI*: −0.010–0.190), and 0.253 (95% *CI*: 0.160–0.345) for the low, medium, and high SRs, respectively. The effect on the soil AK was not significant in the low and medium SRs, but the high SR had a significantly greater effect than the MF. However, there was no significant change in the AK as the number of planting years increased.

### 2.5. Driving Factors and the Relationships between Soil Properties and Vegetable Yield under Different SRs

**Figure 9:**
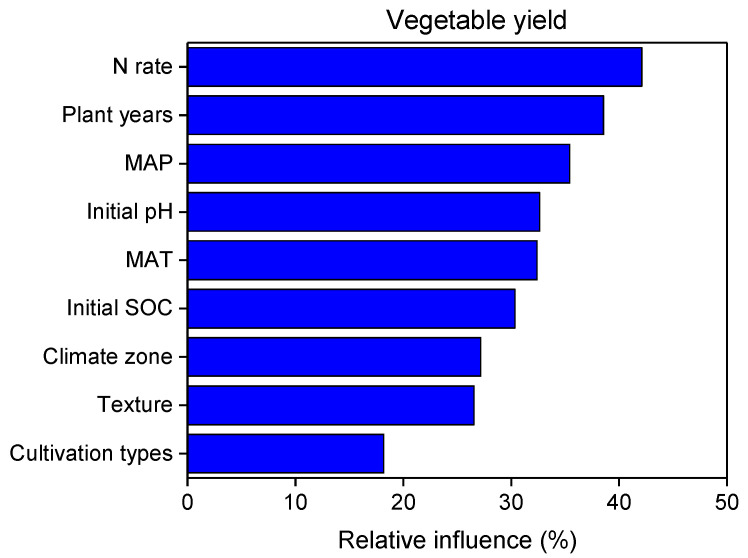
Relative influence of predictor variables on vegetable yield.

The importance of the investigated predictor variables (MAP, MAT, climate zone, texture, initial soil pH and SOC, cultivation type, planting years, and N application) to the vegetable yield and soil properties were explored by a random forest model (Figure 9 and Figure S7). Overall, 83.8% of the variations in vegetable yield could be explained by the nine factors. The N application rate, planting years, MAP, initial soil pH, and MAT were the key influential factors, which explained 42.1%, 38.6%, 35.4%, 32.4%, and 32.4% of the variations in vegetable yield, respectively. Additionally, the initial SOC explained 39.5% of the variations in SOC (81.8%). The N application rate, planting years, and MAT explained 33.9%, 26.6%, and 24.5% of the variations in STN, respectively. The initial SOC was an important factor for the MBC, accounting for 16.2% of the total variation; the climate zone and cultivation types explained 20.2% and 18.1% of the variation in MBN. Additionally, the N application rate, initial SOC, and soil texture were important factors for the AP, accounting for 45.4%, 44.3%, and 41.1%, respectively. Plant years, initial soil pH, and initial SOC explained 38.4%, 33.7%, and 33.1% of the variation in the AK, respectively.

**Figure 10:**
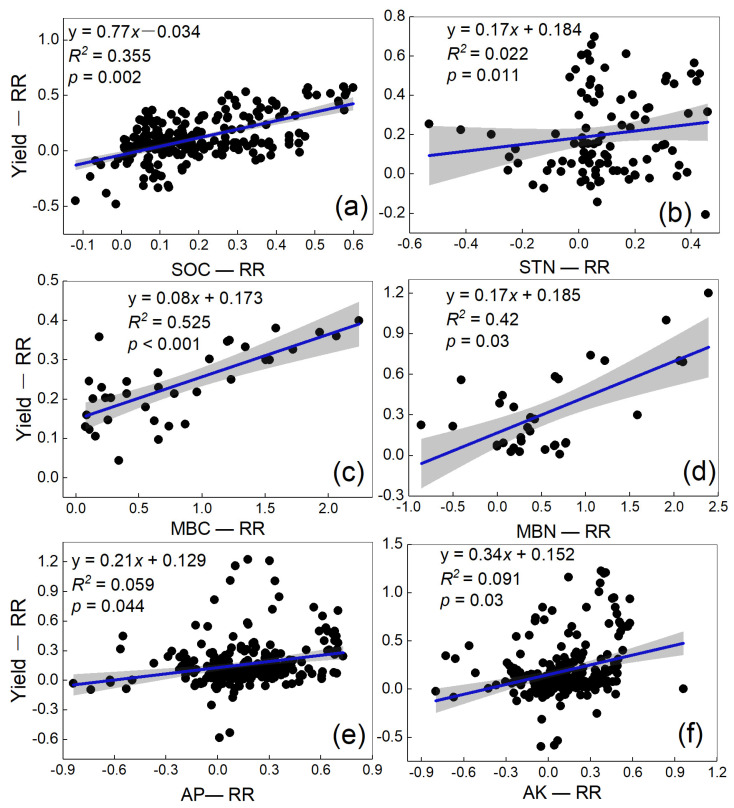
Correlation between vegetable yields and soil property effect values (**a**–**f**). Note: The blue lines indicate the OLS regressions, and the shaded areas indicate the 95% confidence intervals of the fitted regression model. Yield-RR represents the effect size of manure N substitution on vegetable yield compared to mineral fertilizer. SOC−RR, STN−RR, MBC−RR, MBN−RR, AP−RR, and AK−RR represent the effect size of manure N substitution on SOC, STN, MBC, MBN, AP, and AK compared to mineral fertilizer, respectively.

We conducted an OLS regression analysis to examine the relationships between vegetable yields and soil properties (Figure 10). The soil properties were all positively related with the vegetable yield, among which SOC (R^2^ = 0.355, *p* < 0.005); STN (R^2^ = 0.022, *p* < 0.05); MBC (R^2^ = 0.525, *p* < 0.001); MBN (R^2^= 0.42, *p* < 0.05); AP (R^2^ = 0.059, *p* < 0.05); AK (R^2^ = 0.091, *p* < 0.05) and all the regressions were significant. These results show that increasing the soil nutrients had a positive effect on improving the vegetable yields.

## 3. Materials and Methods

### 3.1. Data Collection

Literature on the manure application in vegetable fields in China was collected from Web of Science (https://access.clarivate.com/, accessed on 5 August 2021), Google Scholar (https://scholar.google.com/, accessed on 5 August 2021), and China Knowledge Resource Integrated Database (https://www.cnki.net/old/, accessed on 5 August 2021). The keywords were manure N, vegetable, soil nutrient, and vegetable yield until August 2021. The retrieved articles had to meet the following criteria at the same time: (1) The research area was conducted in mainland China. (2) The study involved the types and rates of N fertilizer, and these were presented. (3) The study had at least one year of field experimentation. (4) The soil sampling depth was 0–20 cm. (5) The mean of the indicator, the standard deviation (SD), and the number of repetitions (n) could be read directly from the table, and graph data were derived using GetData Graph Digitizer 2.26 (http://www.getdata-graph-digitizer.com/). If there were only standard errors (SEs), SD was calculated as SD = SE × √*n* [34]; for studies that did not report SD or SE (accounted for 35.4% of all datasets), the SD was calculated by multiplying the mean by 0.1 [12].

### 3.2. Variables and Grouping

As a result, more than 2176 publications were retrieved by keywords, of which 360 were selected by their title and abstract. Finally, 75 studies with 667 observations on the soil properties (SOC = 275, STN = 103, MBC = 64, MBN = 64, AP = 283, and AK = 285) and vegetable yields (*n* = 434) were compiled into the dataset. To satisfy maximal in-group homogenization [35], the single application of MF was used as the control, and the SR (the ratio of organic N (kg N ha^−1^) over total N application (kg N ha^−1^) was divided into three categories: low (SR ≤ 35%), medium (35% < SR ≤ 70%), and high (SR > 70%) [36].

From the last selected studies, we also recorded the experimental basis (location longitude, and latitude), climatic conditions (mean annual precipitation (MAP), mean air temperature (MAT), and climate zone), initial soil properties (initial soil pH, SOC content, and texture), and management practices (cultivation types, planting years, and N application rate). The following are the specific categories of each parameter we used in this study: MAP (≤600, 600–1000, and >1000 mm); MAT (≤10, 10–15, and >15 °C); climatic zone (temperate and subtropical) [29]; pH (acidic (≤6), neutral (6–8), and alkaline (>8)); SOC (≤10, 10–20, and >20 g kg^−1^); soil texture (sandy, loamy, and clayey) [37]; vegetables (cabbage, small rape, lettuce, etc.), fruits, and pod vegetables (potatoes, beans, cucumbers, etc.); cultivation types (open-air and greenhouse); planting years (≤3, 3–7, and >7 years); and N application rate (fruit and pod vegetables, ≤200, 200–400, and >400 kg N ha^−1^ and leafy vegetables, ≤150, 150–300, and >300 kg N ha^−1^) [36].

### 3.3. Data Analysis

The SR (%) was calculated as follows:

(1) $$SR=\frac{MN}{TN}\times 100$$

where *MN* and *TN* are the rates of organic N (kg N ha^−1^) and total N application (kg N ha^−1^), respectively.

### 3.4. Meta-Analysis

The natural log of the response ratio (ln *R*) was calculated as the effect size [38], representing the effects of manure N substitution ratio:

(2) $$\ln\;R=\ln(\frac{X_{T}}{X_{C}})$$

where *X_C_* is the mean value of soil properties or vegetable yield under chemical fertilizer alone (CK), and *X_T_* represents the mean value of soil properties or vegetable yield under the low, medium, and high SRs, respectively. Experimental sites were considered as a random effect factor when the same experimental site contributed more than one observation in the specific dataset. The variance of ln *R* was determined as follows:

(3) $$v=\frac{SD_{T}{}^{2}}{n_{T}x_{T}{}^{2}}+\frac{SD_{C}{}^{2}}{n_{C}n_{C}{}^{2}}$$

where *SD_T_* and *SD_C_* are the *SDs* of the variables in the MF and manure N substitution treatments, respectively. *n_T_* and *n_C_* are the sample sizes of the variables in the mineral fertilization and manure N substitution treatments, respectively. The weighted effect sizes were calculated as:

(4) $$\ln R_{++}=\frac{\sum \left(w_{i}\times \ln R_{i}\right)}{\sum w_{i}}$$

where ln *R_i_* and *w_i_* are the effect size and corresponding weight of each comparison of the soil properties or vegetable yield. The weight was calculated by the inverse of *v* from Equation (3).

(5) w=1/v

Mean effect sizes and their 95% confidence intervals (*CIs*) were generated through bootstrapping (5000 iterations) [39]:

(6) $$95\%CI=\ln R_{++}\pm 1.96S_{\ln R_{++}}$$

Meta-analysis can determine the significance of the result according to the 95% *CI*. The effects of SRs were considered significant compared with the control (MF) if the *CI* did not overlap with zero. The percent change to quickly account for the response of SR was calculated as:

(7) $$E=[\exp(\ln R_{++})-1]\times 100\%$$

The significance of the heterogeneities between groups (Qb) and within groups was applied by using the Chi-square test. A *p*-value of Qb < 0.05, for instance, represents the significant heterogeneity of the effect size between groups of individual explanatory variables. Qb ≤ 35 indicates that the heterogeneity between treatments is acceptable; 35 < Qb ≤ 70 indicates that there is moderate heterogeneity between treatments, and Qb > 70 indicates that the heterogeneity between treatments is unacceptable [40].

### 3.5. Statistical Analysis

A mixed-effects model using the rma.mv function of the R package metafor and a Wald-type test was used to assess whether treatment effects were statistically different between experimental categories [41]. In addition, we chose climatic conditions, initial soil properties, and management practices as predictor variables, and the boosted regression tree (BTR) analysis using the random forest package (version 4.6–14) in R version 4.1.3 [42] was used to predict the vegetable yield and soil properties. An ordinary least squares (OLS) regression analysis was conducted and plotted using the OriginPro2017 (OriginLab, Northampton, MA, USA) to determine the relationship between changes in soil properties and vegetable yields in soils with SR. Statistical analysis and significance testing were performed using SPSS software 26.0 (SPSS Inc., Chicago, IL, USA). Sigmaplot 10.0 (Systat, San Jose, CA, USA) was used for graphing.

## 4. Discussion

### 4.1. Effect of Manure N Substitution Ratio on Vegetable Yield

Previous studies have found that when the SR was too low or too high to maintain the maximum N use efficiency, it resulted in a decrease in vegetable yields [7]. A meta-analysis carried out in Chinese rice fields showed that the yield was optimal when the SR was 20%, and when the SR exceeded this ratio, the effect would still increase with the SR, but the degree of increase gradually decreased [17]. Our study found that compared with the application of the MF, the vegetable yield increased by 18.6% under the mode of a medium SR of 35–70%, which was significantly higher than those of the low and high SRs in this study, and the SR was also slightly higher than that of Ding [17]. This discrepancy might be because manure N contains more nutrient elements. Compared with grain crops, vegetables demand more nutrient elements in addition to N. Manure N is also rich in organic components, such as carbohydrates, proteins, lipids, and organic acids, making it provide more nutrient elements for vegetable growth [43]. The purpose of a synergistic yield could be achieved by manure N substitution to the MF [44].

Crop yield is affected by many factors, including climate factors, initial soil properties, management practices, etc. [4,24,34]. Our study found that the vegetable yield of the same SR increased gradually with the increase in MAP. This result is consistent with that of [12]. In the same SR, high temperature had a greater impact on yield. When the MAT was ≥15 °C and the climate zone was subtropical, the vegetable yield was the highest, which might be attributable to the fact that warm temperatures increased the rate of vegetable development and yield [45]. Manure N application could provide more available nutrients in subtropical climate zones than in cold temperature regions [46], which was beneficial to the increase in yield.

The initial soil properties are also an important aspect affecting yield. It has been reported that the addition of manure N has a two-way regulating effect on soil pH [12,47], so it is possible to regulate the soil pH to a range suitable for better crop growth and improved yield. In this study, both in initial acidic (pH ≤ 6) or alkaline (pH ≥ 8) soils, the vegetable yield increased with the SR, but when the soil pH was no longer a limiting factor for crop growth, meaning the initial soil pH was 6–8, the nutrient availability had become a key factor in determining yield. This indicates that in acidic (pH ≤ 6) or alkaline (pH ≥ 8) soils, the SR should be appropriately increased to ensure a stable yield. In neutral soil conditions, the SR needs to take nutrient availability into consideration. Similarly, when the initial SOC was <10 g kg^−1^, the vegetable yield increased with the increase in the SR, indicating that the increase in the SR could increase the level of soil fertility [24], which in turn affects vegetable production. However, when the SOC was ≥10 g kg^−1^, the effect value under the medium SR was the largest, which might have a significant relationship with the effectiveness of manure. Soil texture usually determines soil aeration and nutrient retention, which in turn affects yield [48,49]. Our meta-analysis confirmed that compared with the clayey and sandy soils, the yields of the three SRs of the silty soils all showed an increasing trend, and the yield under the medium SR increased the most. This indicates that the application of manure N on silty soil is more conducive to increasing vegetable yield.

In our analysis, the effect value of each SR was higher under open-air cultivation than in greenhouse cultivation, and the yield reduction appeared in the high SR with greenhouse cultivation. This result indicates that the manure N application effect was higher in open-air cultivation than in greenhouse cultivation, which might be related to the high multi-cropping index and the excessive accumulation of nutrients under greenhouse cultivation [7,50], and the degree of response to manure N was not as great as that in open-air cultivation. There were differences in yields among the planting years in the three time periods. When the planting years were less than 3, the high SR would reduce the yield. This might be because the release of nutrients from manure N is a slow process, and the high SR does not release adequate nutrients in a short time to meet the needs of vegetable growth [7], resulting in a reduction in the vegetable yield. At the same time, when the planting years were more than 7, the degree of vegetable yield increase gradually decreased with the increase in SR, but the yield effect value of each SR showed an increasing trend. This also reflects that the long-term application of manure N could ensure stable yields [24]. For the three N application categories, the three SRs showed a trend of increasing yield effect value under the condition of medium N application, and this indicated that the medium N application rate could maintain good yield stability. Therefore, it is recommended that the N application rate of leafy vegetables should be controlled at the medium N application level of 150–300 kg N ha^−1^, and the N application rate of fruit and pod vegetables should be maintained at 200–400 kg N ha^−1^. Meanwhile, the SR of 35% < SR ≤ 70% is the best.

### 4.2. Effect of Manure N Substitution Ratio on Soil C and N in Vegetable Fields

The application of manure N could increase the SOC content by directly inputting C or indirectly increasing the underground C input. Therefore, the SR is an important aspect of the effectiveness of increasing the SOC content [24,51]. Ren et al. [13] showed that about 29% of the C input in manure N remains in the soil, and another source of increased SOC is the supply of C through root exudation or crop residues [52]. Our data show that the SOC content was the greatest in the medium SR, with an increase of 28.5%. The SOC content increased with the increase in planting years, and this shows that the long-term application of organic N fertilizer can improve SOC and soil quality [4]. On one hand, this is because the medium and high SRs could directly input more C sources than the single application of MF and the low SR [14]. On the other hand, compared with the high SR, the medium SR produced a higher crop biomass (such as roots and crop residues) and higher yields [49], simultaneously enhancing the below-ground C input, so that the SOC content was the highest in the medium SR. This result indicates that the medium SR was a reasonable ratio to increase SOC.

The addition, the manure N could improve the NUE, while reducing ammonia volatilization and N leaching losses, which can keep more N in the soil and increase the content of STN [7,17]. Compared with MF, the N supply capacity of manure N was relatively slow under the SRs, and the mineralization rate and N fixation capacity of soils will be different under different SRs of the same manure N type [53]. A meta-analysis study in Chinese croplands showed that soil STN increased by 16% under manure N application compared with a single application of MF [12]. Our study found that the different SRs resulted in different increases in soil STN. Compared with the low and high SRs, the medium SR had the largest average effect size of STN, and the range of STN increases also fluctuated the most, reaching 0.8–41.7% under the medium SR, and after 7 years of planting, the high SR reduced the increase in the STN content. This could be attributable to the fact that N in the soil under the low SR was easily lost through ammonia volatilization and N leaching, so that STN could not be preserved in the soil [7]. While in the high SR, the availability of N in manure N was slightly lower than that of MF, which could not provide usable N for the soil in the short term, and the N fixation capacity of the soil would also be greatly reduced [54]. Overall, our findings confirm that the medium SR could reduce N loss and increase soil N availability, which resulted in the largest increase in STN content.

Soil MBC and MBN are important indicators of soil fertility [16]. A recent meta-analysis showed that the application of manure N in global croplands increased MBC and MBN by 88 ± 6.3 and 84 ± 11%, respectively [47]. In our analysis, the soil MBC and MBN contents increased with the increase in the SR, and they increased by 203.4% and 119.3% in the high SR, respectively. In addition, this study shows that manure N fertilizer can improve the microbial activity of vegetable soil with the increase in planting years. These results confirm that the soil microbial activity in vegetable fields was significantly higher than that of general cropland under the SR. The increase in nutrient availability and the improvement of the microbial growth after the application of manure N simultaneously resulted in a substantial increase in MBC and MBN [55] and thus enhanced biological nitrogen fixation [26].

### 4.3. Effect of Manure N Substitution Ratio on Soil-Available Phosphorus and Potassium in Vegetable Fields

Many studies reported that the application of manure N improved the soil’s physicochemical properties, such as soil aeration, porosity, and soil pH through C input, which was beneficial to the soil’s nutrient balance and improved nutrient availability [4,24,55]. The AP and AK, essential soil nutrients for vegetable growth, were also significantly increased with the manure N application. When it comes to AP, one of the most easily fixed elements in soil, its deficiency is a severe issue for vegetable production in China [15]. The meta-analysis results of Du et al. [12] showed that soil AP increased by 16% in Chinese croplands after the application of manure N. The results of AP and AK under different substitution rate modes showed that the heterogeneity index (Qb) was less than 35, suggesting that our results are robust. Our study found that the increased range of AP was 9.8–28.9% under the three SRs. The rhizosphere effect of manure N significantly increased the AP availability and accelerated the conversion of inactive AP to AP [56]. The long-term application of manure N fertilizer may also cause the accumulation of P. Unlike soil AP, AK is not the main limiting factor for vegetable production in China, but a high rate of potassium fertilizer is still applied to ensure stable crop yields [56]. In our meta-analysis, there was no significant difference in the impact on AK under the low and medium SRs compared with the single application of the MF. The increase growth range of soil AK was 3.5–28.8% with the three SRs. Only in the high SR was the soil AK content significantly higher than that of the MF, which indicated that the supply of potassium in farmland soil in China was in surplus. Our results confirm that the difference of SRs will cause a great difference in soil nutrient availability. It is necessary to formulate reasonable SR strategies to maintain nutrient availability. The increase in nutrient availability can reduce farmers’ dependence on MFs.

A higher soil quality generally increases vegetable productivity. A global meta-analysis showed that manure N application increased SOC and nutrient (TN, AP, and AK) contents and microbial activity, with positive effects on crop yields [4,30,57]. Cai et al. [4] reported that increases in SOC and soil nutrient storage had a positive effect on the yield under the application of manure N. Our study shows that higher SOC, STN, MBC, MBN, and soil-available nutrients (AP and AK) were all positively related with vegetable yields (*p* < 0.05). Therefore, substituting MF with manure N can ensure an increase in yield through the increased nutrient availability and soil quality.

### 4.4. Uncertainties in Quantifying the Ratio of Substituting Mineral Fertilizers with Manure N

Our meta-analysis summarized and synthesized the ratio of substituting mineral fertilizers with manure N on vegetable yield and soil properties and identified the key factors in China. However, we are aware that our meta-analysis has some important limitations. First, due to the limited data, most of the ratio of substituting mineral fertilizers with manure N in the studies is based on N content, and the substitution rate of C, S, P, etc. and other indicators is rarely recorded. When substituting fertilizer N for manure N, C, S, P, etc. are applied. Manure treatments received these other elements, but the fertilized treatment did not. Our study did not consider the effects of other elements. The contents of N and other nutrients (P, K, etc.) in the manure remained uncertain for the results, and this lack of information limited us to predict the comprehensive responses of soil properties to different manure fertilizer SRs, which is also a common problem encountered in most meta-analyses [58]. Therefore, we recommend to judge the impact degree of the manure application rate and its SRs on soil properties and crop yields before a meta-analysis in future studies. If manure the application rate and its SRs are low, the effects of other nutrients in the manure on the soil properties and crop yields can be ignored. Otherwise, we need to determine the differences in other nutrients between mineral fertilizers and manure fertilizers by analyzing their impact on the soil properties and crop yields and then eliminate or reduce the effects caused by the other nutrients existing in the manure fertilizer.

## 5. Conclusions

In this study, a meta-analysis was used to explore the differences in topsoil (0–20 cm) nutrients and the effects of different SRs on vegetable yields. The results show that compared with the single application of MF, the SOC and STN improved the most with the medium SR, and the soil MBC, MBN, and AP contents all increased with the increase in the SR. The soil AK was significantly increased only in the high SR. Soil nutrients have a positive correlation with vegetable yield. The medium SR had the greatest effect on the yield improvement, with an increase of 18.6%. A random forest analysis showed that the N application rate, planting years, and MAP were the most important factors influencing vegetable yield, while climate zone, soil texture, and cultivation types had the weakest effects. Our results suggest that the SR in the range of 35–70% is recommended as the optimal organic replacement ratio for Chinese vegetable fields. This SR range can improve the effectiveness of soil nutrients while maintaining a high level of vegetable yields.

## Figures and Tables

**Table 1:** Divided groups in the categorical meta-analysis of vegetable yield response to different substitution ratio (SR) compared to mineral fertilizer (MF).

Index	Low SR (*n* = 168)		Medium SR (*n* = 163)		High SR (*n* = 103)	
	Qb	*p*	Qb	*p*	Qb	*p*
MAP	5.82	0.188	6.10	0.102	5.62	0.103
MAT	4.80	0.209	1.68	0.492	3.87	0.166
Climate zone	2.58	0.203	0.12	0.807	3.37	0.075
pH	48.00	0.001	37.42	0.03	44.97	0.001
SOC	64.29	0.001	13.18	0.042	20.31	0.004
Soil texture	24.83	0.007	12.75	0.037	13.56	0.014
Vegetable type Cultivation type	4.61 10.59	0.341 0.012	7.92 7.78	0.161 0.014	5.66 1.57	0.266 0.243
Planting years	36.00	0.002	2.27	0.668	1.98	0.606
N application	13.98	0.009	5.78	0.125	4.04	0.19

Note: MAP, mean annual precipitation; MAT, mean annual temperature; SOC, soil organic carbon; Low SR, SR ≤ 35%; Medium SR, 35% < SR ≤ 70%; High SR, SR > 70%; Qb, between-group heterogeneity; *p* < 0.05 indicates a significant difference.

## Data Availability

Not applicable.

## Supplementary Materials

The following supporting information can be downloaded at: https://www.mdpi.com/article/10.3390/plants12040964/s1, Figure S1: Effect of organic manure substitution ratios (SR) on soil organic carbon (SOC, g/kg) with different climatic conditions, initial soil properties, and management practices. Figure S2: Effect of manure N substitution ratios (SR) on soil total nitrogen (STN, g/kg) with different climatic conditions, initial soil properties, and management practices. Figure S3: Effect of manure N substitution ratios (SR) on microbial biomass carbon (MBC, mg/kg) with different climatic conditions, initial soil properties, and management practices. Figure S4: Effect of manure N substitution ratios (SR) on microbial biomass nitrogen (MBN, mg/kg) with different climatic conditions, initial soil properties, and management practices. Figure S5: Effect of manure N substitution ratios (SR) on available phosphorus (AP, mg/kg) with different climatic conditions, initial soil properties, and management practices. Figure S6: Effect of manure N substitution ratios (SR) on available potassium (AK, mg/kg) with different climatic conditions, initial soil properties, and management practices. Figure 7S: Relative influence of predictor variables on soil nutrients (SOC, Soil organic carbon (g/kg; STN, soil total N; MBC/MBN, microbial biomass C/N; AP, available phosphorus; AK, available potassium). Table S1: Divided groups in the categorical meta-analysis of soil nutrient (soil organic carbon(g/kg), SOC; soil total nitrogen(g/kg), STN; microbial biomass carbon (C) and nitrogen (N)(mg/kg), MBC/N; available phosphorus and potassium (mg/kg), (AP/AK)) responses to different manure N SRs compared to mineral fertilizer. Table S2: Number of observations, mean, median, and 95% confidence intervals for soil properties under different substitution ratio (SR).
