# Supplementary material for: Effects of the Ratio of Substituting Mineral Fertilizers with Manure Nitrogen on Soil Properties and Vegetable Yields in China: A Meta-Analysis
Source: Plants (Basel). 2023 Feb 20;12(4):964. doi: 10.3390/plants12040964 (PMC9959476; doi:10.3390/plants12040964)
Supplement: Supplementary file 1 [file plants-12-00964-s001.zip › plants-2013371-supplementary.pdf]

Effects of the ratio of substituting mineral fertilizers with manure nitrogen on soil properties and vegetable yields in China: A meta-analysis

Shaobo Wang[1], Rui Lv [1] Xinhua Yin[2], Puyu Feng[1], and Kelin Hu[1,*]

1 College of Land Science and Technology, China Agricultural University, Key Laboratory of Arable Land Con-servation (North China), Ministry of Agriculture, Beijing, 100193, China

2 Department of Plant Sciences, University of Tennessee, Knoxville, TN 37996, USA

* Correspondence: hukel@cau.edu.cn

Table S1. Divided groups in the categorical meta-analysis of soil nutrient (soil organic carbon(g/kg), SOC; soil total nitrogen(g/kg), STN; microbial biomass carbon (C) and nitrogen (N)(mg/kg), MBC/N; available phosphorus and potassium (mg/kg), (AP/AK)) responses to different manure N SRs compared to mineral fertilizer.

SOC

Index	Low SR(n = 106)		Medium SR(n = 89)		High SR(n = 79)	
	Qb	p	Qb	p	Qb	p
MAP	32.83	0.001	31.36	0.001	19.40	0.024
MAT	28.64	0.001	19.21	0.01	15.21	0.047
Climate zone	22.24	0.001	32.68	0.001	7.53	0.087
pH	18.19	0.011	17.44	0.042	10.41	0.025
SOC	21.11	0.039	10.02	0.028	28.96	0.009
Soil texture	10.98	0.024	11.91	0.028	16.28	0.101
Cultivation types	33.17	0.001	10.00	0.025	6.04	0.026
Planting years	2.95	0.016	35.46	0.003	18.35	0.047
N application	17.96	0.004	28.52	0.004	22.59	0.016

Note: MAP, mean annual precipitation(mm); MAT, mean annual temperature(℃); SOC, Soil organic carbon(g/kg). Low SR, SR ≤ 35%; Medium SR,35%＜ SR ≤ 70%; High SR, SR > 70%; Qb, between-group heterogeneity. ; $p < 0.05$ indicates a significant difference.

STN

Index	Low SR(n = 37)		Medium SR(n = 22)		High SR(n = 42)	
	Qb	p	Qb	p	Qb	p
MAP	52.47	0.001	8.90	0.281	0.37	0.913
MAT	17.59	0.152	26.45	0.006	4.36	0.288
Climate zone	7.23	0.237	0.96	0.679	3.40	0.176
pH	49.10	0.048	0.59	0.918	12.70	0.081
SOC	17.43	0.161	10.41	0.218	1.07	0.761
Soil texture	23.50	0.021	12.31	0.294	10.02	0.112
Cultivation types	35.03	0.01	24.66	0.026	9.52	0.034
Planting years	13.10	0.41	15.60	0.139	4.30	0.47
N application	31.18	0.021	1.50	0.726	8.33	0.093

Note: MAP, mean annual precipitation(mm); MAT, mean annual temperature(℃); SOC, Soil organic carbon(g/kg). Low SR, SR ≤ 35%; Medium SR,35%＜ SR ≤ 70%; High SR, SR > 70%; Qb, between-group heterogeneity ; $p < 0.05$ indicates a significant difference.

MBC

Index	Low SR(n = 24)		Medium SR(n = 21)		High SR(n = 16)	
	**Qb**	***p***	**Qb**	***p***	**Qb**	***p***
MAP	6.57	0.255	22.80	0.002	8.46	0.114
MAT	23.98	0.004	2.83	0.083	14.41	0.008
Climate zone	6.81	0.108	15.89	0.003	16.35	0.026
pH	7.83	0.084	22.08	0.001	0.91	0.507
SOC	17.71	0.008	0.97	0.565	31.23	0.001
Soil texture	15.23	0.002	4.96	0.171	0.63	0.573
Cultivation types	14.22	0.017	0.01	0.922	5.38	0.111
Planting years	15.46	0.017	11.27	0.024	26.35	0.26
N application	12.18	0.026	0.15	0.683	0.88	0.503

Note: MAP, mean annual precipitation(mm); MAT, mean annual temperature(℃); SOC, Soil organic carbon(g/kg). Low SR, SR ≤ 35%; Medium SR,35%＜ SR ≤ 70%; High SR, SR > 70%; Qb, between-group heterogeneity. ; $p < 0.05$ indicates a significant difference.

MBN

Index	Low SR(n = 24)		Medium SR(n = 18)		High SR(n = 14)	
	**Qb**	***p***	**Qb**	***p***	**Qb**	***p***
MAP	1.283	0.741	1.461	0.379	1.990	0.535
MAT	26.575	0.001	1.750	0.323	18.648	0.005
Climate zone	1.063	0.447	0.859	0.520	21.489	0.064
pH	15.408	0.006	0.859	0.512	5.995	0.050
SOC	26.350	0.003	2.819	0.443	2.908	0.192
Soil texture	15.264	0.021	37.524	0.004	5.995	0.059
Cultivation types	4.992	0.120	13.799	0.001	21.489	0.090
Planting years	24.412	0.020	36.888	0.005	44.841	0.000
N application	27.991	0.003	3.017	0.162	26.647	0.021

Note: MAP, mean annual precipitation(mm); MAT, mean annual temperature(℃); SOC, Soil organic carbon(g/kg). Low SR, SR ≤ 35%; Medium SR,35%＜ SR ≤ 70%; High SR, SR > 70%; Qb, between-group heterogeneity. ; $p < 0.05$ indicates a significant difference.

AP

Index	Low SR(n = 114)		Medium SR(n = 94)		High SR(n = 72)	
	**Qb**	***p***	**Qb**	***p***	**Qb**	***p***
MAP	22.168	0.034	1.340	0.785	4.892	0.100
MAT	25.558	0.014	12.656	0.139	12.683	0.006
Climate zone	4.764	0.266	0.104	0.866	9.335	0.009
pH	13.251	0.297	23.936	0.067	2.789	0.422
SOC	31.332	0.053	16.981	0.139	5.489	0.166
Soil texture	11.869	0.347	23.525	0.019	9.104	0.040
Cultivation types	1.375	0.568	18.138	0.015	0.031	0.874
Planting years	27.588	0.067	18.384	0.006	6.610	0.118
N application	3.141	0.686	26.355	0.012	13.538	0.005

Note: MAP, mean annual precipitation(mm); MAT, mean annual temperature(℃); SOC, Soil organic carbon(g/kg). Low SR, SR ≤ 35%; Medium SR,35%＜ SR ≤ 70%; High SR, SR > 70%; Qb, between-group heterogeneity. ; $p < 0.05$ indicates a significant difference.

AK

Index	Low SR(n = 118)		Medium SR(n = 93)		High SR(n = 73)	
	**Qb**	***p***	**Qb**	***p***	**Qb**	***p***
MAP	27.494	0.002	32.042	0.001	1.549	0.354
MAT	15.495	0.073	7.579	0.103	1.825	0.264
Climate zone	3.883	0.246	0.019	0.921	1.099	0.189
pH	24.734	0.001	4.511	0.340	0.403	0.858
SOC	0.533	0.969	10.056	0.109	0.084	0.986
Soil texture	17.574	0.106	5.807	0.273	0.378	0.897
Cultivation types	7.200	0.118	0.043	0.873	1.532	0.125
Planting years	10.069	0.305	23.000	0.001	0.744	0.766
N application	1.836	0.751	0.363	0.890	0.120	0.898

Note: MAP, mean annual precipitation(mm); MAT, mean annual temperature(℃); SOC, Soil organic carbon(g/kg). Low SR, SR ≤ 35%; Medium SR,35%＜ SR ≤ 70%; High SR, SR > 70%; Qb, between-group heterogeneity. ; p < 0.05 indicates a significant difference.

Table S2. Number of observations, mean, median, and 95% confidence intervals for soil properties under different substitution ratio(SR).

Soil properties	Treatment	*n*	Mean	Median	95% *CI*
SOC(g kg^{-1})	MF	102	10.7c	12.2	6.2-20.3
	SR≤35%	106	15.1b	15.8	10.3-24.9
	35%＜SR≤70%	89	16.3a	16.1	4.5-27.9
	SR＞70%	80	16.4a	16.0	9.2-27.5
STN(g kg^{-1})	MF	42	1.0c	1.1	0.5-2.4
	SR≤35%	38	1.3b	1.7	0.5-3.3
	35%＜SR≤70%	22	1.8a	1.2	0.2-3.1
	SR＞70%	43	1.4b	1.5	0.6-2.3
MBC(mg kg^{-1})	MF	20	19.1d	12.9	2-53.9
	SR≤35%	25	29.6c	15.9	1.5-123
	35%＜SR≤70%	22	78.4b	89.2	11.4-158.2
	SR＞70%	17	100.3a	67.0	21-293
MBN(mg kg^{-1})	MF	20	8.5d	1.8	0.6-53
	SR≤35%	25	15.9c	11.8	0.3-68.1
	35%＜SR≤70%	22	25.3b	22.5	2.7-78.1
	SR＞70%	17	29.7a	24.4	2.3-69.4
AP(mg kg^{-1})	MF	103	115.0b	86.3	15.4-396.3
	SR≤35%	115	138.6a	114.9	17.4-337.6
	35%＜SR≤70%	95	139.4a	110.7	17.4-403
	SR＞70%	73	137.8a	107.8	19-420
AK(mg kg^{-1})	MF	99	241.0b	172.3	17-820
	SR≤35%	119	245.6b	229.4	64.5-602
	35%＜SR≤70%	93	266.9a	265.0	14.4-721
	SR＞70%	73	260.3a	236.5	16.8-912

Note: SOC, soil organic carbon; STN, soil total nitrogen; MBC, microbial biomass carbon; MBN, microbial biomass nitrogen; AP, available phosphorus; AK, available potassium. MF, mineral fertilizers; n=number of measurements; Means in a row followed by different letters are significantly different at $p < 0.05$.

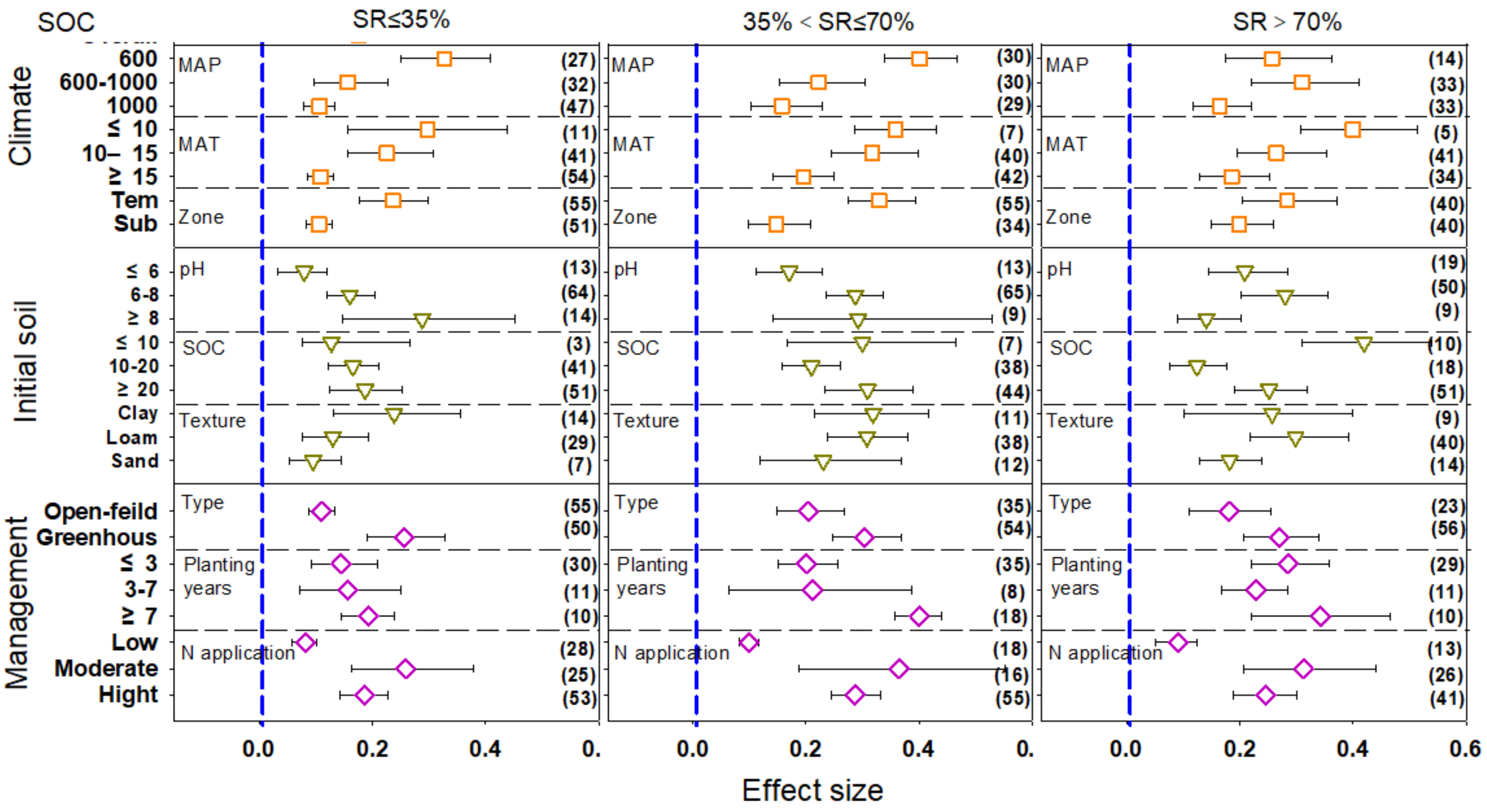

Figure S1. Effect of organic manure substitution ratios (SR) on soil organic carbon (SOC, g/kg) with different climatic conditions, initial soil properties, and management practices. Note: MAP is mean annual precipitation (mm), MAT is mean annual temperature(℃); SOC, Soil organic carbon(g/kg).Tem is temperate climate zone, Sub is subtropical climate zone, and Type is cultivation type. Dashed line indicates effect size = 0.

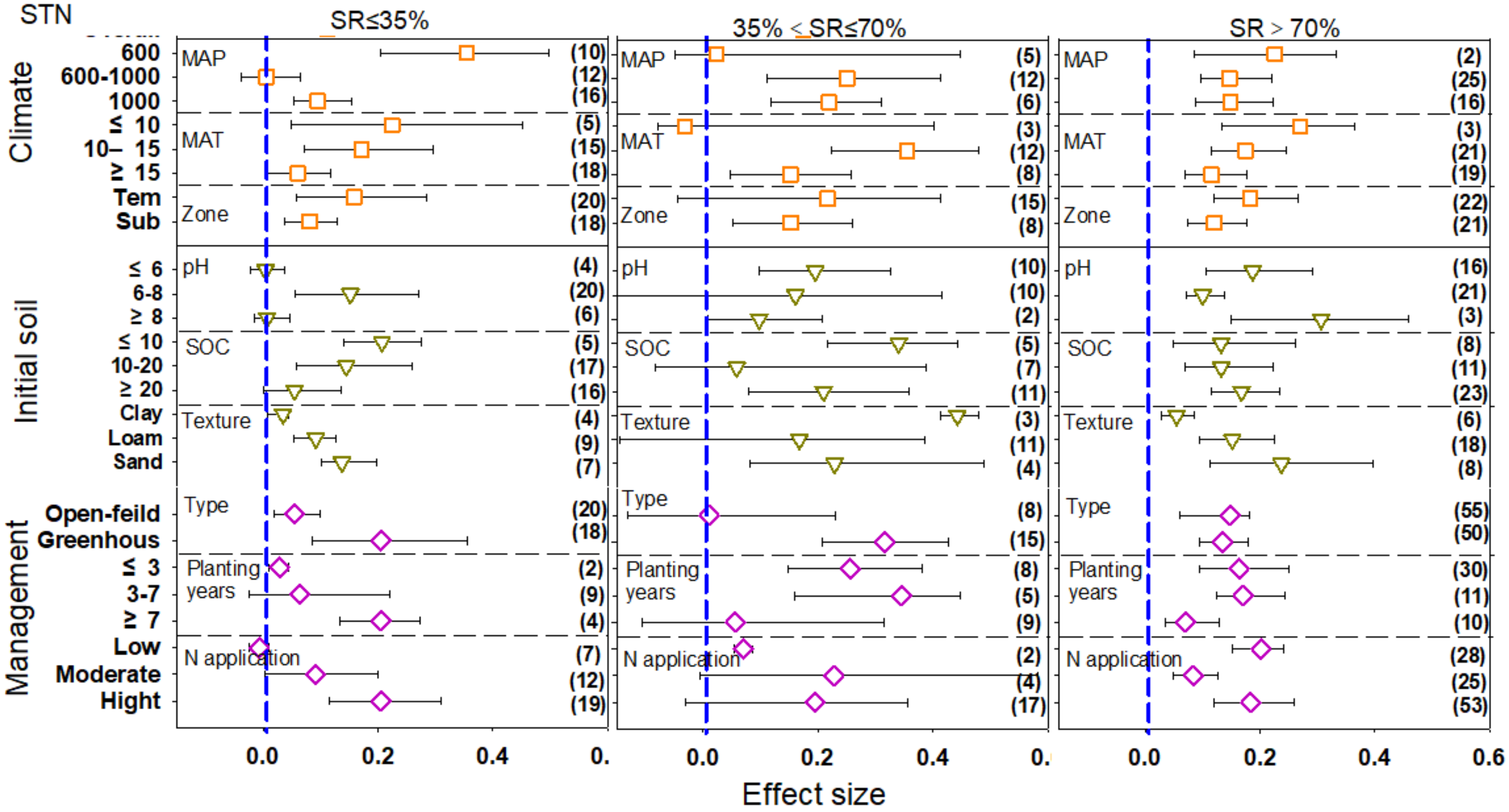

Figure S2. Effect of manure N substitution ratios (SR) on soil total nitrogen (STN, g/kg) with different climatic conditions, initial soil properties, and management practices. Note: MAP is mean annual precipitation(mm), MAT is mean annual temperature (℃); SOC, Soil organic carbon(g/kg). Tem is temperate climate zone, Sub is subtropical climate zone, and Type is cultivation type. Dashed line indicates effect size = 0.

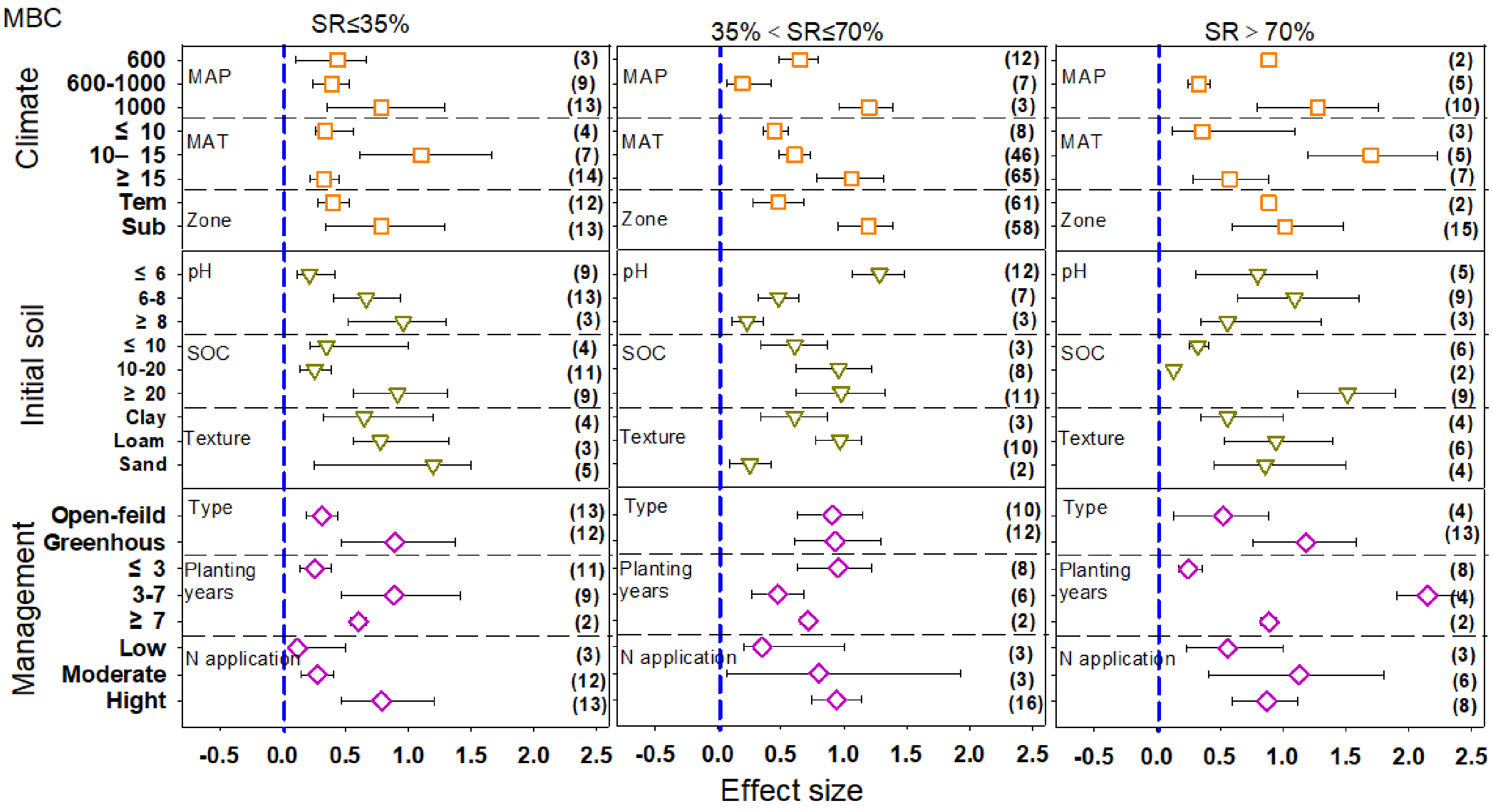

Figure S3. Effect of manure N substitution ratios (SR) on microbial biomass carbon (MBC, mg/kg) with different climatic conditions, initial soil properties, and management practices. Note: MAP is mean annual precipitation(mm), MAT is mean annual temperature(℃), SOC, Soil organic carbon(g/kg). Tem is temperate climate zone, Sub is subtropical climate zone, and Type is cultivation type. Dashed line indicates effect size = 0.

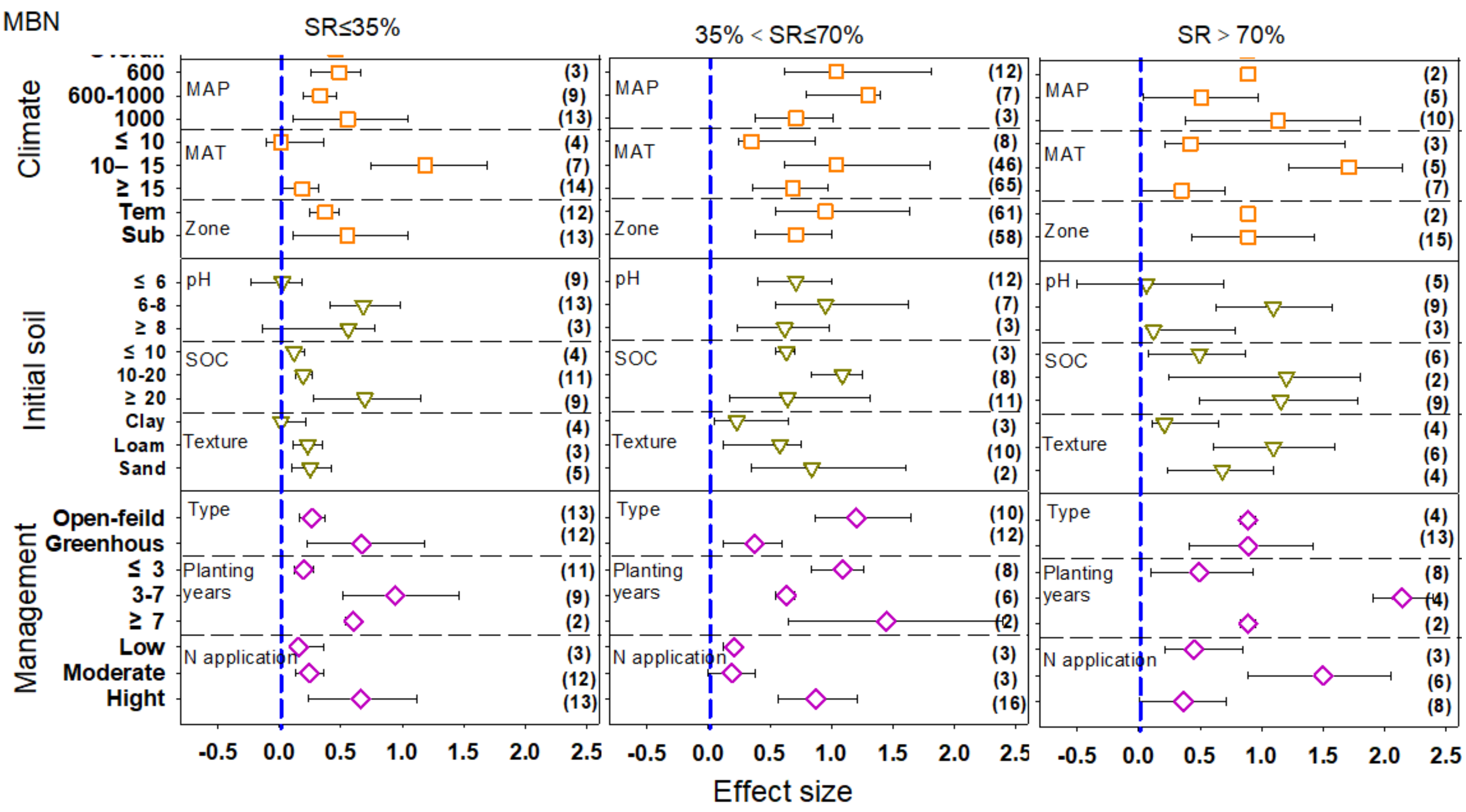

Figure S4. Effect of manure N substitution ratios (SR) on microbial biomass nitrogen (MBN, mg/kg) with different climatic conditions, initial soil properties, and management practices. Note: MAP is mean annual precipitation(mm), MAT is mean annual temperature(℃), SOC, Soil organic carbon(g/kg). Tem is temperate climate zone, Sub is subtropical climate zone, and Type is cultivation type. Dashed line indicates effect size = 0.

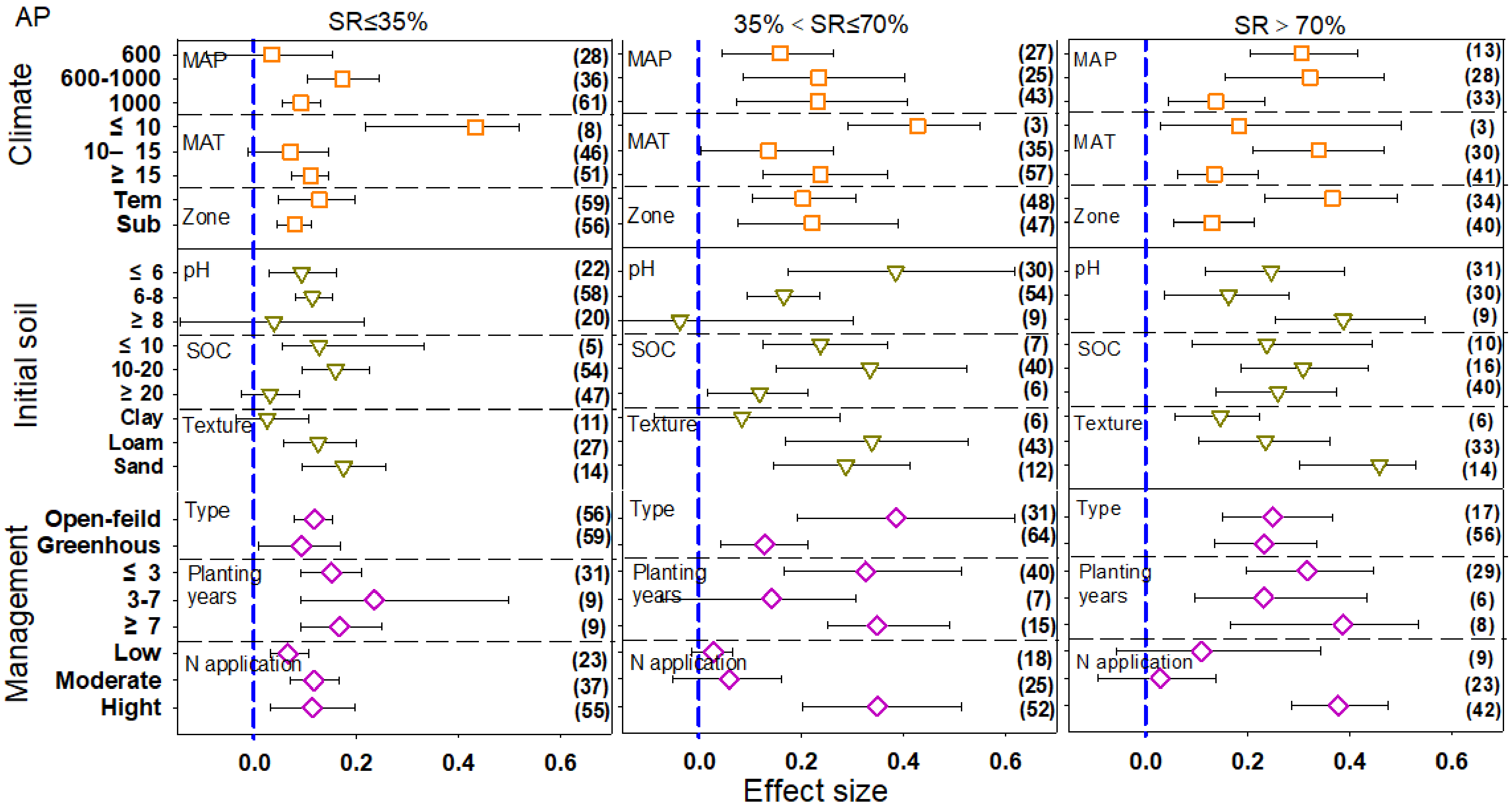

Figure S5. Effect of manure N substitution ratios (SR) on available phosphorus (AP, mg/kg) with different climatic conditions, initial soil properties, and management practices. Note: MAP is mean annual precipitation(mm), MAT is mean annual temperature(°C); SOC, Soil organic carbon(g/kg).Tem is temperate climate zone, Sub is subtropical climate zone, and Type is cultivation type. Dashed line indicates effect size = 0.

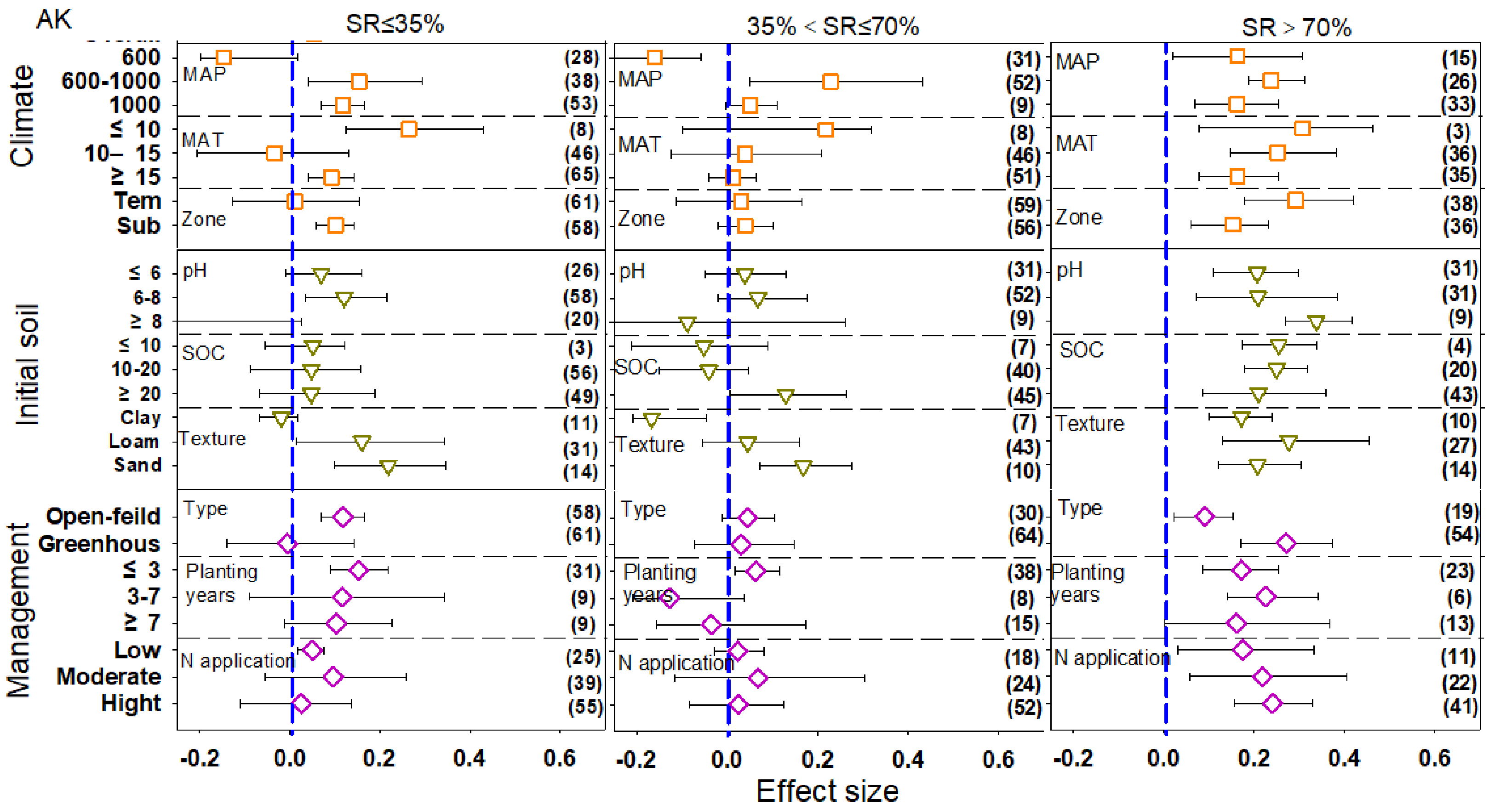

Figure S6. Effect of manure N substitution ratios (SR) on available potassium (AK, mg/kg) with different climatic conditions, initial soil properties, and management practices. Note: MAP is mean annual precipitation(mm), MAT is mean annual temperature(℃); SOC, Soil organic carbon(g/kg). Tem is temperate climate zone, Sub is subtropical climate zone, and Type is cultivation type. Dashed line indicates effect size = 0.

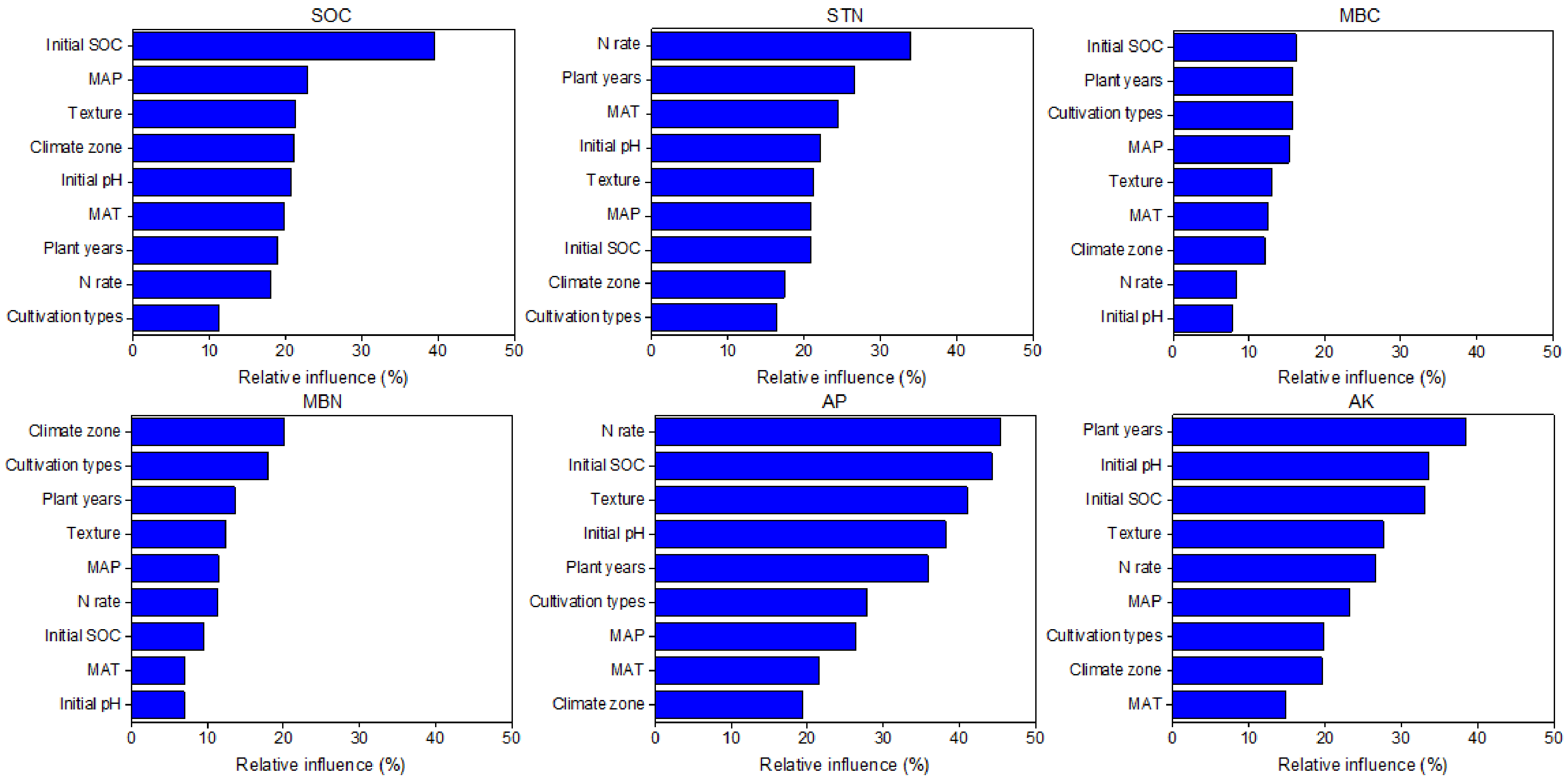

Figure S7. Relative influence of predictor variables on soil nutrients (SOC, Soil organic carbon(g/kg; STN, soil total N; MBC/MBN, microbial biomass C/N; AP, available phosphorus; AK, available potassium). Note: MAP is mean annual precipitation(mm), MAT is mean annual temperature(℃).
